# The Quality of MitraClip™ Content on YouTube

**DOI:** 10.7759/cureus.43881

**Published:** 2023-08-21

**Authors:** Bradley M Nus, Trey Sledge, Kylie Wu, Christian S Saunders, Wissam Khalife

**Affiliations:** 1 Cardiology, University of Texas Medical Branch, Galveston, USA; 2 Cardiology, Texas College of Osteopathic Medicine, Fort Worth, USA

**Keywords:** social media, cardiology, quality, accreditation, patient education, youtube, mitraclip

## Abstract

Objective

YouTube (YouTube LLC, San Bruno, California, United States) is used as a primary resource for many patients looking to gain healthcare knowledge. Recently, YouTube made efforts to increase the quality of posted content by accrediting trusted healthcare sources. With an increasing emphasis being placed on minimally invasive options, this study was done to investigate the quality of YouTube videos on MitraClip™ (Abbott Laboratories, Chicago, Illinois, United States) with respect to patient education.

Methods

YouTube was searched using the keyword “MitraClip”. A total of 66 videos were evaluated, with 32 of those videos being included for final analysis after applying exclusionary criteria. Three independent reviewers separately scored the videos using the Global Quality Scale. Likes, dislikes, views, comments, and dates of upload were also recorded. Two-tailed t-tests were used to determine statistical significance.

Results

MitraClip videos on YouTube proved to be of medium quality, receiving an average Global Quality Scale score of 3.39. When stratified by the new YouTube accreditation process, those with accreditation had a significantly higher Global Quality Scale score of 4.11, while non-accredited videos had an average Global Quality Scale score of 3.12 (p<0.01). Shorter and more patient-friendly videos were also significantly lower in quality (p<0.05).

Conclusion

The YouTube accreditation process has demonstrated initial success at regulating the quality of MitraClip content, thereby reducing the spread of misinformation. However, this progress is undermined by the lack of unique videos present on the platform. Increasing the amount of original content about MitraClip may allow viewers to diversify their educational sources and ultimately gain a better understanding of the procedure.

## Introduction

Internet usage in people of all age groups has dramatically increased in the past decade, with many of these online searches pertaining to healthcare information for themselves or loved ones [1,2]. Rather than navigating laboriously through articles, individuals often watch videos, as they are an easy way to gather information with minimal effort required from the viewer [3]. With over 3.4 billion monthly viewers, YouTube (YouTube LLC, San Bruno, California, United States) is the leading video platform and contains an immense catalog of healthcare content [4,5]. However, creators and the content they produce are not subject to any peer review prior to upload, often leading to highly inconsistent video content quality [6]. This facilitates the spread of misinformation that may influence patient decisions and strain the physician-patient relationship [7-11]. In response to these concerns, YouTube recently implemented a new accreditation process that clearly labels videos from trusted healthcare sources [12]. Trusted sources of information include certain healthcare or government organizations, educational institutions, or public health departments. These sources were determined to be reliable based on principles developed by the National Academy of Medicine and verified by the World Health Organization (WHO). Even the latest studies analyzing the quality of medical information on YouTube are yet to investigate this recent policy change.

Currently, over two million Americans are afflicted by mitral regurgitation, a condition that is often overlooked and undertreated [13,14]. MitraClip™ (Abbott Laboratories, Chicago, Illinois, United States) aims to alleviate this burden by providing a less invasive option for patients who are poor candidates for open heart surgery [15,16]. With more emphasis being placed on minimally invasive options in recent years, it is important that patients are properly educated on the MitraClip procedure [17]. Notably, MitraClip content on YouTube was previously studied in a 2015 abstract [18]. However, due to recent policy changes and the rapidly evolving nature of online information, follow-up is necessary. Therefore, the purpose of this study was to define the educational landscape of MitraClip videos on YouTube with respect to patient education.

## Materials and methods

Search strategy

**Figure 1:**
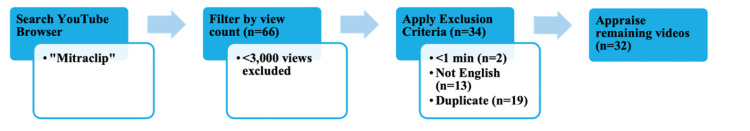
Methodology for YouTube* video selection *YouTube LLC, San Bruno, California, United States

The study methodology was based on that of multiple previous studies regarding online healthcare content [19,20]. YouTube (www.youtube.com) was searched for the key term “MitraClip” on November 5, 2021. To avoid customized search results, the inquiry was done on Google Chrome (Google LLC, Mountain View, California, United States) in "incognito" mode. The search results were then set to "filter by view count" on YouTube’s advanced search parameters. This organizes videos from most viewed to least viewed, thereby ensuring the relevance of the videos included in the study. Videos with <3,000 views were excluded, leaving 66 videos for analysis. Next, content that was less than one minute (n=2), not in English (n=13), or a duplicate (n=19) was also excluded from the study. Videos less than one minute were excluded as these did not contain sufficient information to accurately assess. Content that was not narrated or captioned in English was excluded as this is the universally accepted language in many countries [21]. This left a total of 32 videos suitable for final analysis. Search strategies are summarized in Figure 1.

Video assessment

Each video was independently analyzed by three authors (BN, KW, TS) to minimize personal bias. All three reviewers were third-year medical students who were well-versed in the MitraClip procedure. Content quality was judged using the Global Quality Scale (GQS), a five-point DISCERN score adapted to video content, as described in previous studies [22-25]. Each question is answered with a yes or no, awarding the video 1 point or 0 points, respectively (Table 1). After all five questions are answered, the GQS score is calculated by adding the total points received. This creates a range of 0-5 with 0 representing the lowest quality videos and 5 representing the highest quality videos (Table 2).

**Table 1:** Global Quality Scale (GQS) criteria used to score MitraClip videos on YouTube

Quality of information (1 point for Yes, 0 points for No)
1. Are the aims clear and achieved?
2. Are reliable sources of information used?
3. Is the information balanced and unbiased?
4. Are additional sources of information provided for patient reference?
5. Are areas of uncertainty mentioned?

**Table 2:** Assessment of MitraClip video quality based on Global Quality Scale (GQS) score

GQS score	Description
1	Poor quality, misleading, clear bias
2	Subpar quality, may be misleading, some bias
3	Moderate quality, may contain bias, not all information accurate
4	Good quality, generally unbiased, missing some information and references
5	Excellent quality, accurate, unbiased

Descriptive video characteristics including likes, dislikes, comments, view count, duration, modality, and date of upload were recorded. The new YouTube accreditation process was used to separate videos that were accredited versus those that were not. Videos were also stratified by the presence or absence of a board-certified physician substantiating the information at any point. Finally, video modality was used to categorize content into "Patient Friendly" or "Clinician Friendly" groups. Patient accounts of their experience with MitraClip, physician interviews, and animations were included in the "Patient Friendly" group. These required minimal health literacy to comprehend and were designed to educate the general public on the procedure. In contrast, academic presentations and live surgeries were categorized into the "Clinician Friendly" group. Prior medical knowledge was essential to understand this content as it was oriented toward practicing physicians looking to further their education on MitraClip.

View ratio and a like-to-dislike ratio were used to assess video popularity, as noted in previous studies [26]. The view ratio was calculated as: $\frac{V}{U}$, where V= total number of views; U = number of days since upload. The like-to-dislike ratio was calculated as $\frac{L}{D}$, where L=total number of likes, D=total number of dislikes.

Statistical analysis

Statistical analysis was performed with IBM SPSS Statistics for Windows, Version 26.0 (2019; IBM Corp., Armonk, New York, United States). The normality of the data was tested using the Shapiro-Wilks test. The intraclass correlation coefficient (ICC) was used to assess score reliability among the three independent reviewers. Pairwise comparisons between average GQS scores and video categories were evaluated using the Mann-Whitney U test. A p-value of <0.05 was considered statistically significant. Continuous variables were reported as mean and standard deviation while categorical variables were reported as frequency. The Pearson correlation coefficient was used to analyze relationships between GQS score and video characteristics. Numerical results were rounded to two decimal places.

## Results

MitraClip videos on YouTube proved to be of moderate quality, receiving an average GQS score of 3.39. However, of the 66 videos evaluated, 51.52% (34/66) were excluded, with the majority of those being duplicates of the same exact animation (n=19). MitraClip videos were an average of 1,661.81 days (4.55 years) old and garnered an average of 19,712 views per video. Videos about MitraClip were, on average, 920.72 seconds or 15.35 minutes in length. This is about four minutes longer than the average 11.7-minute video length on YouTube as a whole [27]. The view and like-to-dislike ratios were, on average, 16.44 and 12.35, respectively. Videos with likes and dislikes disabled were excluded from the like-to-dislike ratio calculation (n=2). Additional descriptive data is provided in Table 3.

**Table 3:** General characteristics of MitraClip* YouTube** videos *Abbott Laboratories, Chicago, Illinois, United States **YouTube LLC, San Bruno, California, United States

	Mean (±SD) (n=32)	Range (min - max)
Days Since Upload	1,661.84 ± 1,103.58	39 - 4,384
View Count	19,712 ± 35,715.11	3,189 - 165,356
Duration (sec)	920.72 ± 1,276.72	66 - 5361
Likes	78.67 ± 141.85	0 - 584
Dislikes	5.73 ± 9.1	0 - 39
Comments	5.65 ± 11.94	0 - 49
Like-to-Dislike Ratio	12.35 ± 8.52	1.32 - 26.55
View Ratio	16.44 ± 23.97	1.1 - 98.5
GQS Score	3.396 ± 1.28	0 - 5

**Figure 2:**
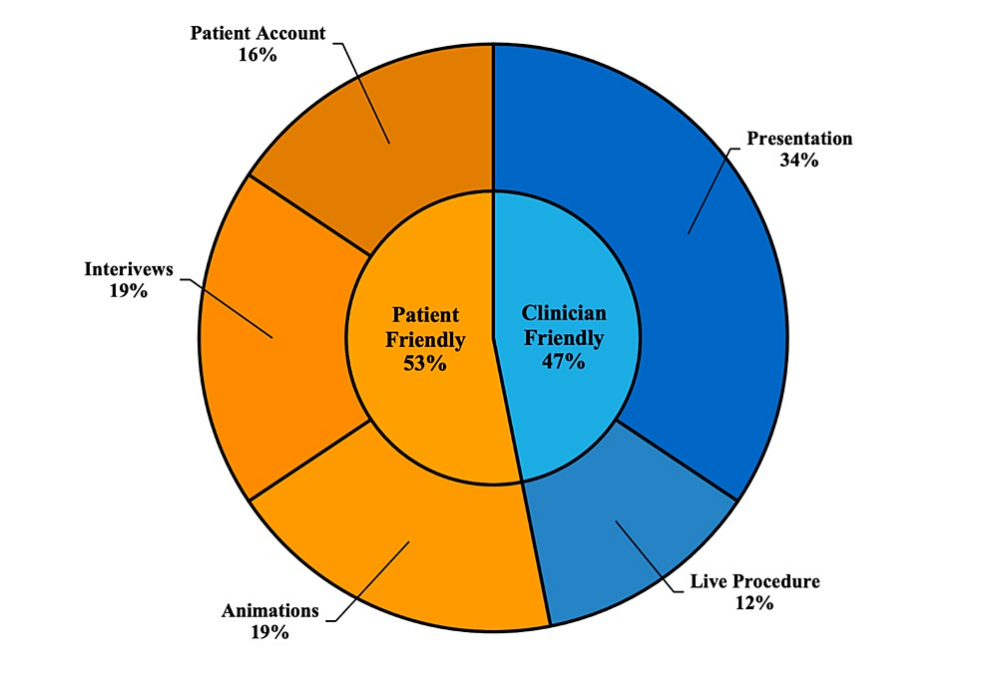
Video content stratified by presumed target audience

When categorized by target audience, 17 videos (53%) were patient friendly and 15 videos (47%) were clinician friendly. Patient friendly videos included six animations (19%), six physician interviews (19%), and five patient accounts (16%). Clinician friendly videos included 11 academic presentations (34%), and four live MitraClip procedures (12%) (Figure 2).

**Figure 3:**
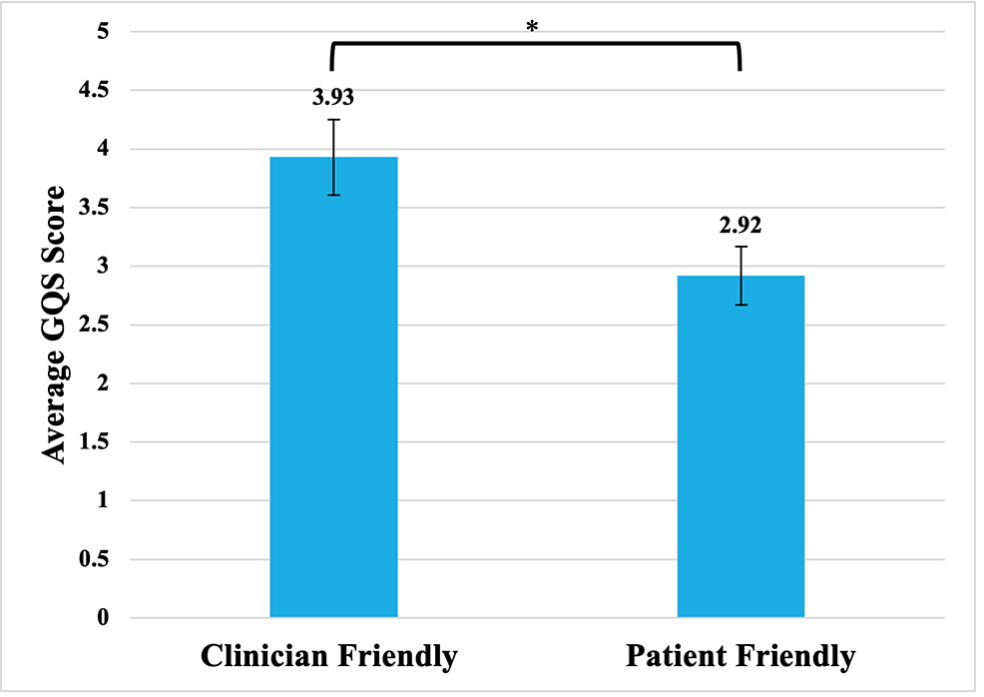
Video GQS score when grouped by target audience GQS: global quality scale

Patient friendly videos had an average GQS Score of 2.92. This was significantly lower than clinician friendly videos, which had an average GQS Score of 3.93 (p<0.05) (Figure 3).

**Figure 4:**
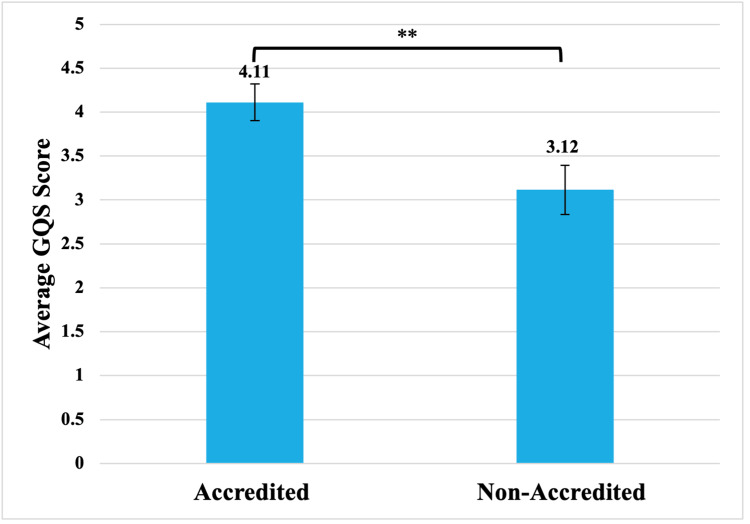
Video GQS score when stratified by the YouTube accreditation process GQS: global quality scale

Only nine (28.13%) MitraClip videos were labeled as reliable by the newly implemented YouTube accreditation process, while the remaining 23 (71.87%) videos were not. MitraClip videos with accreditation had a significantly higher GQS score of 4.11, while non-accredited videos had an average GQS score of 3.12 (p<0.01) (Figure 4).

Shorter videos were also found to be of significantly lower quality. Videos under five minutes (n=15) in length received an average GQS score of 2.84, while those longer than five minutes (n=17) had an average GQS score of 3.88 (p<0.05). 

No significant correlation was found between average GQS Scores and days since upload (R2 =0.07). There was no significant difference in average GQS scores between videos with view ratios higher than 6 (n=15) and those lower than 6 (n=17) (p=0.2). This was also the case when videos were separated by those above and below a view ratio of 10 (p=0.18). When stratified by accreditation status, videos did not have significantly different view ratios (p=0.56). No significant difference was found when comparing average GQS scores between groups with (n=24) and without (n=8) a physician present to substantiate the claims made in the video at any point (p=0.08). The ICC for GQS scores was 0.92, which indicated excellent agreeability among the three independent reviewers [28].

## Discussion

YouTube is the most prominent video platform on the internet and an increasingly popular source of healthcare information [2,4]. However, the quality of the content is highly variable as videos are not subject to any peer review process prior to upload [6]. Credible videos uploaded to the site empower patients to make informed healthcare decisions and facilitate strong patient-physician relationships [29]. Alternatively, healthcare misinformation may leave patients confused or confidently incorrect on subjects vital to their health and well-being. Therefore, the risk of encountering misinformation on the platform may outweigh the potential benefits this site provides as a source of patient education. Mitigating the effects of this misinformation may be done in two ways: (1) increased content moderation by YouTube; and (2) improved physician understanding of the platform. In theory, content moderation would be an optimal way to combat online misinformation. For instance, the coronavirus disease 2019 (COVID-19) outbreak in 2020 led to a plethora of online misinformation regarding the topic to the point that the WHO referred to it as an "infodemic" [30]. To counteract this, WHO began to promote evidence-based messages and collaborated with major online presences including Google (Google LLC), Amazon (Amazon.com, Inc., Seattle, Washington, United States), and Instagram (Meta Platforms, Inc., Menlo Park, California, United States) to take down inaccurate information regarding the pandemic. These efforts were able to mitigate a substantial amount of misinformation in circulation [31]. However, due to the rapid spread of news across platforms, the idealistic situation of a completely misinformation-free online realm is yet to be achieved [32].

Additionally, due to the commercial interests of YouTube, there are numerous difficulties in ensuring adequate quality content moderation [33]. Therefore, it is justified to start with what can be controlled. Providers may not have control over YouTube policy, but they can become well-versed in the online space to effectively guide patients toward credible videos and rectify frequently encountered misconceptions.

In response to these growing concerns, numerous studies have been published to define the educational value of healthcare content on YouTube [24,26]. However, there remains a paucity of research regarding videos about MitraClip, a life-changing treatment option for those with severe mitral regurgitation. This study found that among the sparse catalog of MitraClip YouTube content, videos were of moderate educational quality, with clinician-centered and accredited videos receiving the highest average GQS scores.

The insufficient quantity of original MitraClip videos on YouTube undermines patients’ ability to properly educate themselves on the subject. Over half (56%) of the videos excluded from analyses were duplicates of the same exact MitraClip animation. This is troublesome as previous studies have highlighted the importance of diverse visual representations of data when learning [34]. Viewers who struggled to understand the MitraClip procedure with initial attempts on the platform may become frustrated or discouraged upon realizing the lack of alternative videos. Furthermore, this paucity of unique MitraClip content was also noted in a 2015 abstract, indicating a static trend [18]. The solution to this problem is simple: more original MitraClip videos must be uploaded to YouTube. This change could be instigated by physicians or medical institutions and would empower patients to make better-informed healthcare decisions.

The educational value of MitraClip videos is not only hindered by content quantity, but content comprehensibility as well. This becomes apparent when stratifying videos by the target audience. Clinician-friendly videos contained academic presentations and live procedures that were clearly intended for viewers with a background in healthcare. Although this content contained significantly higher quality information than patient-friendly videos, they were often unsuitable for educating those with low health literacy. One possible reason for this discrepancy may be a lack of trained professionals present in patient-friendly content. However, stratifying by the presence or absence of a board-certified physician in the video failed to produce significant results when compared to average GQS scores (p=0.08). An alternative explanation is the oversimplification of the MitraClip procedure in patient-friendly videos. To appeal to a broader audience, video creators often omit important details, thus lowering the video’s educational quality. One such detail that was often omitted from patient-friendly videos was information regarding areas of uncertainty such as the relative risks and benefits of the MitraClip procedure. These omissions are troublesome as individuals with low health literacy are highly susceptible to online misinformation [35]. Therefore, patient-friendly content, although easier to understand, may leave viewers with a highly biased and unrealistic view of the MitraClip procedure. In the future, more emphasis must be placed on creating MitraClip videos that maintain high educational quality without sacrificing comprehensibility.

In response to growing concerns about online healthcare misinformation, YouTube implemented a new accreditation process on January 26, 2021 [12]. Accredited videos are only uploaded from reputable sources (Harvard University, Mayo Clinic, American Public Health Association, etc.) and remain pinned to the top of relevant search results, thus increasing the likelihood of viewers consuming this information first [36]. Despite the importance of this policy change, no previous articles have investigated its efficacy. This study found that accredited MitraClip videos contained significantly higher-quality information than their non-accredited counterparts. These videos were generally unbiased, included references for further inquiry, and addressed details such as alternative treatment options and areas of uncertainty. Therefore, clinicians can inform patients to prioritize accredited videos when researching MitraClip outside of their limited appointment time. Currently, only nine MitraClip videos are accredited, emphasizing the need for continued expansion of this policy to ensure a diverse catalog of high-quality MitraClip content.

Besides accreditation status, several additional video characteristics were compared with GQS scores to elucidate factors related to content quality. Of these, only video duration yielded significant results. On average, content over five minutes in length was higher in quality than content shorter than five minutes. This result is likely because longer videos had ample time to provide a sufficiently detailed explanation of the MitraClip procedure. Therefore, physicians should urge their patients to avoid videos shorter than five minutes to obtain the highest quality information.

Notably, no significant correlation was found between the date of video upload and GQS scores. This indicates a stagnant trend in content quality and emphasizes the need for expansion of the current MitraClip YouTube catalog. View ratios were not significantly different between videos with and without accreditation status. However, this finding may be due to a lack of sufficient time for accredited videos to gain significantly more views than non-accredited content. Similarly, average GQS scores were not significantly different between videos with high versus low view ratios. Previous studies have found mixed results regarding viewership and content quality [37], indicating that video popularity is not a reliable marker of educational value. This distinction should be communicated to patients as YouTube suggests videos based on view count and other engagement statistics [38].

Limitations

The primary limitation of this study was the small sample size as noted previously. Less stringent exclusionary criteria and an increased quantity of MitraClip YouTube content are necessary for more robust analyses in the future. Additionally, an unavoidable limitation of this study is the profit-driven business model of YouTube as a platform. YouTube's main source of revenue is through advertisements on their videos and is thereby subjected to influences from their investors who place an emphasis on profit [39]. Every time an advertisement is viewed or clicked, YouTube charges advertisers between $0.10 to $0.30 [40]. Therefore, YouTube’s business model may leave its content vulnerable to biases by prioritizing video watchability over video educational quality. Another constraint of this study was the method of data collection. Video metrics were taken at a single point in time; however, this does not accurately reflect the rapidly evolving nature of online healthcare information. Further limitations include a lack of generalizability to non-English speaking countries, and exclusion of healthcare content present on sites other than YouTube. Finally, only videos above 3,000 views were investigated, which may have excluded popular content that was recently uploaded.

Future study

This study, to the best of our knowledge, was the first to evaluate the YouTube accreditation process. Future research in this field must account for this policy change to reveal its long-term implications. Furthermore, MitraClip content on popular video platforms besides YouTube, such as TikTok (TikTok Pte. Ltd., Cayman Islands) and Facebook (Meta Platforms LLC), should also be analyzed.

## Conclusions

The YouTube accreditation process has demonstrated initial success at regulating the quality of MitraClip content, thereby reducing the spread of misinformation. However, this progress is undermined by the lack of unique and high-quality patient-friendly videos currently on the platform. Increasing the amount of original, patient-centered content regarding MitraClip may allow viewers to diversify their education sources and ultimately gain a better understanding of the procedure.
